# Fatty Acid Elongation in Non-Alcoholic Steatohepatitis and Hepatocellular Carcinoma

**DOI:** 10.3390/ijms15045762

**Published:** 2014-04-04

**Authors:** Sonja M. Kessler, Yvette Simon, Katja Gemperlein, Kathrin Gianmoena, Cristina Cadenas, Vincent Zimmer, Juliane Pokorny, Ahmad Barghash, Volkhard Helms, Nico van Rooijen, Rainer M. Bohle, Frank Lammert, Jan G. Hengstler, Rolf Mueller, Johannes Haybaeck, Alexandra K. Kiemer

**Affiliations:** 1 Department of Pharmacy, Pharmaceutical Biology, Saarland University, Campus C2 2, 66123 Saarbrücken, Germany; E-Mails: yvette.simon84@gmail.com (Y.S.); pharm.bio.kiemer@mx.uni-saarland.de (A.K.K.); 2 Institute of Pathology, Medical University of Graz, 8010 Graz, Austria; E-Mail: johannes.haybaeck@medunigraz.at; 3 Department of Pharmacy, Pharmaceutical Biotechnology, Saarland University and Helmholtz Institute for Pharmaceutical Research Saarland (HIPS), Helmholtz Centre for Infection Research (HZI), 66123 Saarbrücken, Germany; E-Mails: Katja.Gemperlein@helmholtz-hzi.de (K.G.); rom@mx.uni-saarland.de (R.M.); 4 Systems Toxicology, Leibniz Research Centre for Working Environment and Human Factors (IfADo) at the TU Dortmund, Ardeystr. 67, 44139 Dortmund, Germany; E-Mails: gianmoena@ifado.de (K.G.); cadenas@ifado.de (C.C.); hengstler@ifado.de (J.G.H.); 5 Department of Medicine II, Saarland University Medical Center, Kirrberger Str., 66421 Homburg, Germany; E-Mails: Vincent.Zimmer@uniklinikum-saarland.de (V.Z.); Frank.Lammert@uniklinikum-saarland.de (F.L.); 6 Institute of Pathology, Saarland University, Kirrberger Str., 66421 Homburg, Germany; E-Mails: juliane.pokorny@uniklinikum-saarland.de (J.P.); rainer.bohle@uniklinikum-saarland.de (R.M.B.); 7 Center for Bioinformatics, Saarland University, Campus E2 1, 66123 Saarbrücken, Germany; E-Mails: barghash@bioinformatik.uni-saarland.de (A.B.); volkhard.helms@bioinformatik.uni-saarland.de (V.H.); 8 Department of Molecular Cell Biology, Faculty of Medicine, Vrije Universiteit, Van der Boechorststraat 7, 1081 BT Amsterdam, The Netherlands; E-Mail: nvanrooijen@clodronateliposomes.org

**Keywords:** diethylnitrosamine, ELOVL6, HCC, hepatic steatosis, leptin deficiency, MCD, non-alcoholic fatty liver disease (NAFLD), *ob/ob*

## Abstract

Non-alcoholic steatohepatitis (NASH) represents a risk factor for the development of hepatocellular carcinoma (HCC) and is characterized by quantitative and qualitative changes in hepatic lipids. Since elongation of fatty acids from C16 to C18 has recently been reported to promote both hepatic lipid accumulation and inflammation we aimed to investigate whether a frequently used mouse NASH model reflects this clinically relevant feature and whether C16 to C18 elongation can be observed in HCC development. Feeding mice a methionine and choline deficient diet to model NASH not only increased total hepatic fatty acids and cholesterol, but also distinctly elevated the C18/C16 ratio, which was not changed in a model of simple steatosis (*ob*/*ob* mice). Depletion of Kupffer cells abrogated both quantitative and qualitative methionine-and-choline deficient (MCD)-induced alterations in hepatic lipids. Interestingly, mimicking inflammatory events in early hepatocarcinogenesis by diethylnitrosamine-induced carcinogenesis (48 h) increased hepatic lipids and the C18/C16 ratio. Analyses of human liver samples from patients with NASH or NASH-related HCC showed an elevated expression of the elongase *ELOVL6*, which is responsible for the elongation of C16 fatty acids. Taken together, our findings suggest a detrimental role of an altered fatty acid pattern in the progression of NASH-related liver disease.

## 1. Introduction

Non-alcoholic fatty liver disease (NAFLD) is regarded as the most common liver disorder [1]. Although NAFLD is often an asymptomatic disease and therefore difficult to detect, the prevalence appears to be around 20%–35% of the adult population in Western countries [2–4]. NAFLD/NASH (non-alcoholic steatohepatitis) strongly correlates with characteristics of the metabolic syndrome, such as obesity and diabetes mellitus, and NAFLD/NASH [5–7]. Liver pathogenesis of NAFLD is widely believed to start with simple steatosis, which is characterized by excessive lipid accumulation [2,8]. The progression from simple steatosis to NASH is mediated by the release of inflammatory cytokines [9] and can result in hepatic cirrhosis and finally in hepatocellular carcinoma (HCC) [10]. Due to this inflammatory environment 4% to 27% of individuals with NASH and cirrhosis [11] develop HCC.

There is increasing evidence that in steatosis besides the total amount of accumulated lipids the composition of lipids has an impact on pathophysiology [12,13]. In fact, human NAFLD is characterized by numerous changes in hepatic lipid composition and free fatty acid ratios [14,15]. Viral hepatitis has also been described to lead to strongly altered hepatic lipid content and composition [16–19]. In HCC a decreased stearic acid (C18:0) to oleic acid (C18:1) ratio compared to normal tissue has been reported, suggesting the importance of desaturation in HCC development [20]. Regarding changes in hepatic fatty acid pattern it is important to note that the ELOVL fatty acid elongase 6 (ELOVL6), which catalyzes the elongation of C16 to C18 fatty acids [21], has been shown to promote NASH [22,23]. A role of ELOVL6 in murine NASH-related HCC has recently been suggested, but still remains unclear in human NASH-associated HCC [23].

The aims of our study were to investigate the occurrence of fatty acid elongation in lipid metabolism in different NAFLD mouse models and to elucidate its relevance in human NASH and NASH-associated HCC.

## 2. Results and Discussion

### 2.1. Fatty Acid Elongation in Murine Non-Alcoholic Steatohepatitis (NASH) Is Kupffer Cell Dependent

**Figure 1.**
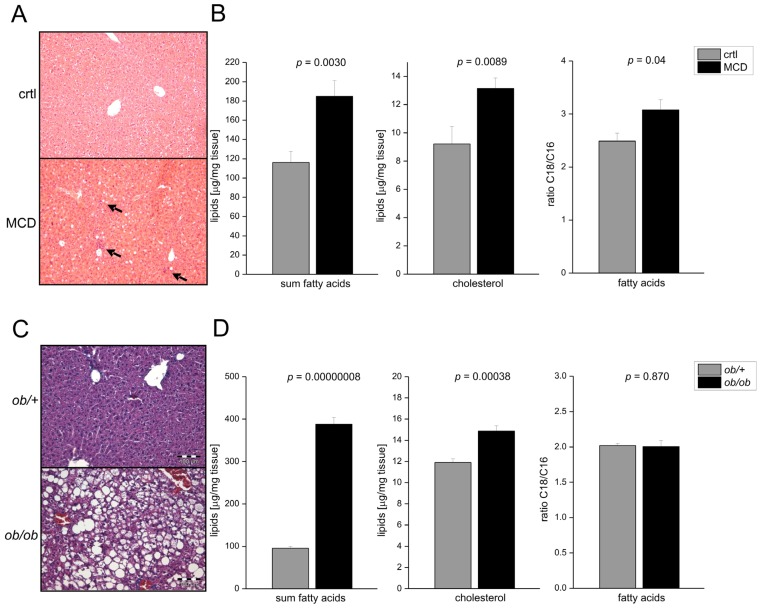
Non-alcoholic steatohepatitis (NASH), but not non-alcoholic fatty liver disease (NAFLD), is accompanied by elevation of C18 over C16. (**A**) Representative liver sections stained with hematoxylin-eosin (HE) from animals fed with either a methionine-choline deficient (MCD) or a control (crtl) diet for 3 weeks (original magnification 200×). Arrows denote inflammatory foci; (**B**) Sum of all hepatic fatty acids, hepatic cholesterol, and ratio of hepatic C18/C16 fatty acids of MCD fed animals compared to ctrl were analyzed by GC-MS (gas chromatography-mass spectrometry) (*n* = 9–10); and (**C**,**D**) Representative HE-stained liver sections (**C**), hepatic fatty acids as well as hepatic cholesterol, and ratio of hepatic C18/C16 fatty acids (**D**) of *ob*/*+* and *ob*/*ob* mice (*n* = 8).

Fatty acid elongation plays a role in murine and human NASH development [22,23]. Feeding mice a methionine-choline deficient diet (MCD) led to increased total fatty acid and cholesterol levels and profound inflammation (Figure 1A,B). Having a closer look at the composition of the fatty acids, we observed that the chain length of fatty acids was different in MCD-fed livers compared to tissues from a control diet: NASH livers exhibited an increased ratio of C18 to C16 fatty acids (Figure 1B). Increased *ELOVL6* mRNA expression has been observed in NASH animal models, such as low-density lipoprotein receptor knockout animals fed a western type diet [23] or a fructose diet [24]. In order to study whether this increased fatty acid elongation from C16 to C18 could also be observed in simple steatosis we investigated the well-established leptin-deficiency (*ob*/*ob*) mouse model [24,25]. As expected, livers of *ob*/*ob* mice showed excessive hepatic lipid accumulation compared to lean controls (Figure 1C,D). However, no distinct inflammation was observed and the ratio of C18 to C16 fatty acids was not changed compared to wild-type animals (Figure 1D). Adipose tissue of *ob*/*ob* mice is known to show inflammation [26]. Concordantly, adipose tissue macrophages of *ob*/*ob* mice exhibit increased *ELOVL6* mRNA expression [27]. Another study reported increased hepatic *ELOVL6* expression in older *ob*/*ob* mice, whereby some of the animals were in a fasted condition [28]. The study does not contain any information on fatty acid composition.

**Figure 2.**
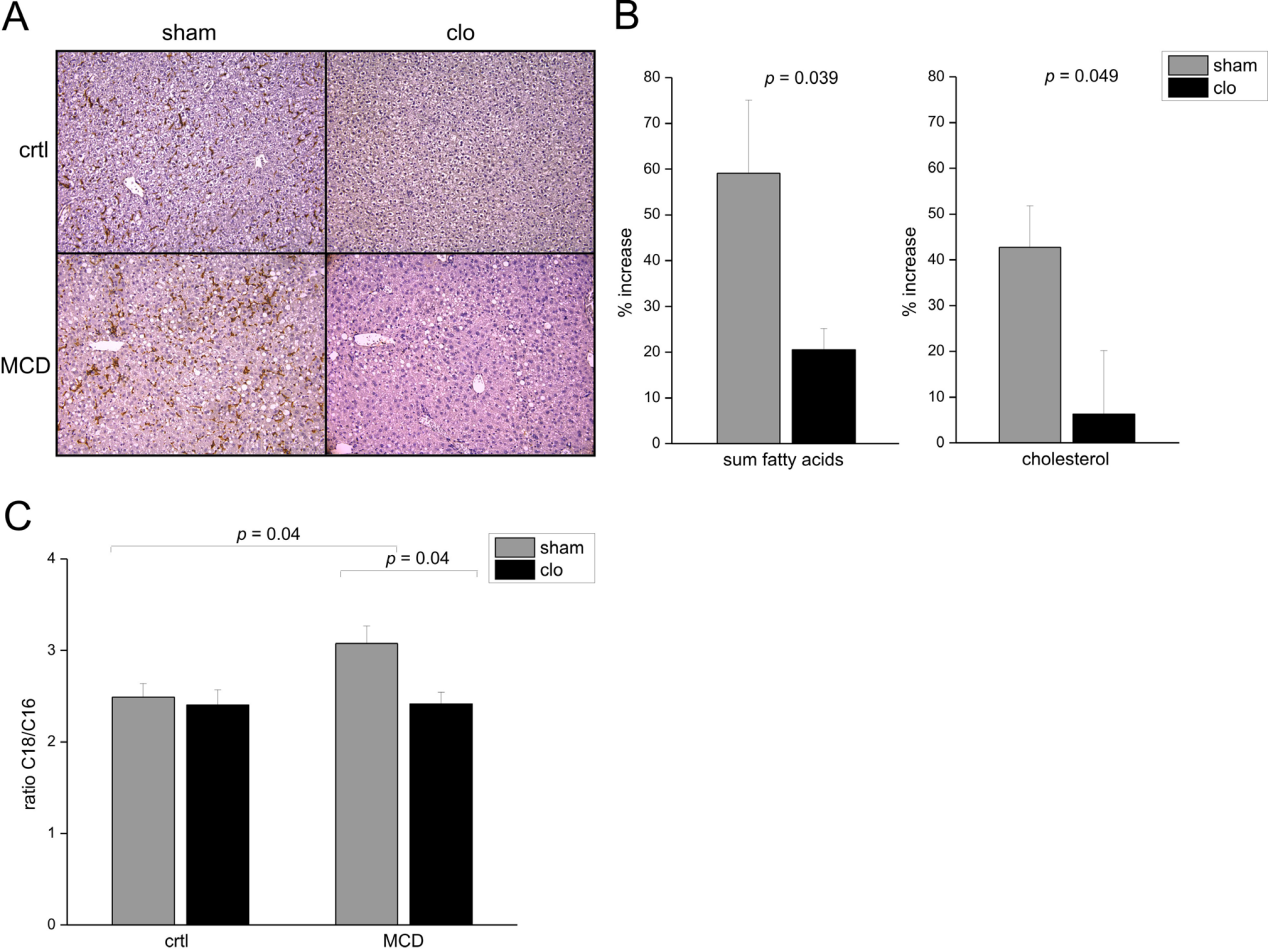
Kupffer cell depletion abrogated elevation of C18 over C16. (**A**) Representative liver sections immunohistologically stained against F4/80 as Kupffer cell marker from animals fed with the respective diet for 3 weeks with simultaneous administration of clodronate (clo) or empty (sham) liposomes (original magnification 200×); and (**B**,**C**) Increase of the sum of all hepatic fatty acids, hepatic cholesterol (**B**), and ratio of hepatic C18/C16 fatty acids (**C**) of MCD fed animals treated with clodronate (clo) or empty (sham) liposomes compared to ctrl analyzed by GC-MS (*n* = 9–10).

Leroux *et al.* reported that lipid storage by Kupffer cells correlates with a pro-inflammatory phenotype in NASH [29]. In order to clarify whether the altered fatty acid elongation in murine NASH is due to co-existing inflammation, we depleted Kupffer cells by clodronate liposomes. This intervention is known to attenuate inflammatory and metabolic events in the MCD model [30]. In line with these findings we observed a strong decrease of hepatic lipid accumulation after Kupffer cell depletion (Figure 2A,B). Besides hepatocytes also macrophages are known to express *ELOVL6* [27], which was shown to be relevant for lipid storage [31]. After Kupffer cell depletion the MCD-induced changes in C18 to C16 fatty acids and cholesterol were completely abrogated (Figure 2A,B). Kupffer cell depletion was confirmed by immunohistochemical F4/80 staining (Figure 2A).

### 2.2. Role of Fatty Acid Elongation in NASH-Related Hepatocellular Carcinoma (HCC) and Human NASH

**Figure 3.**
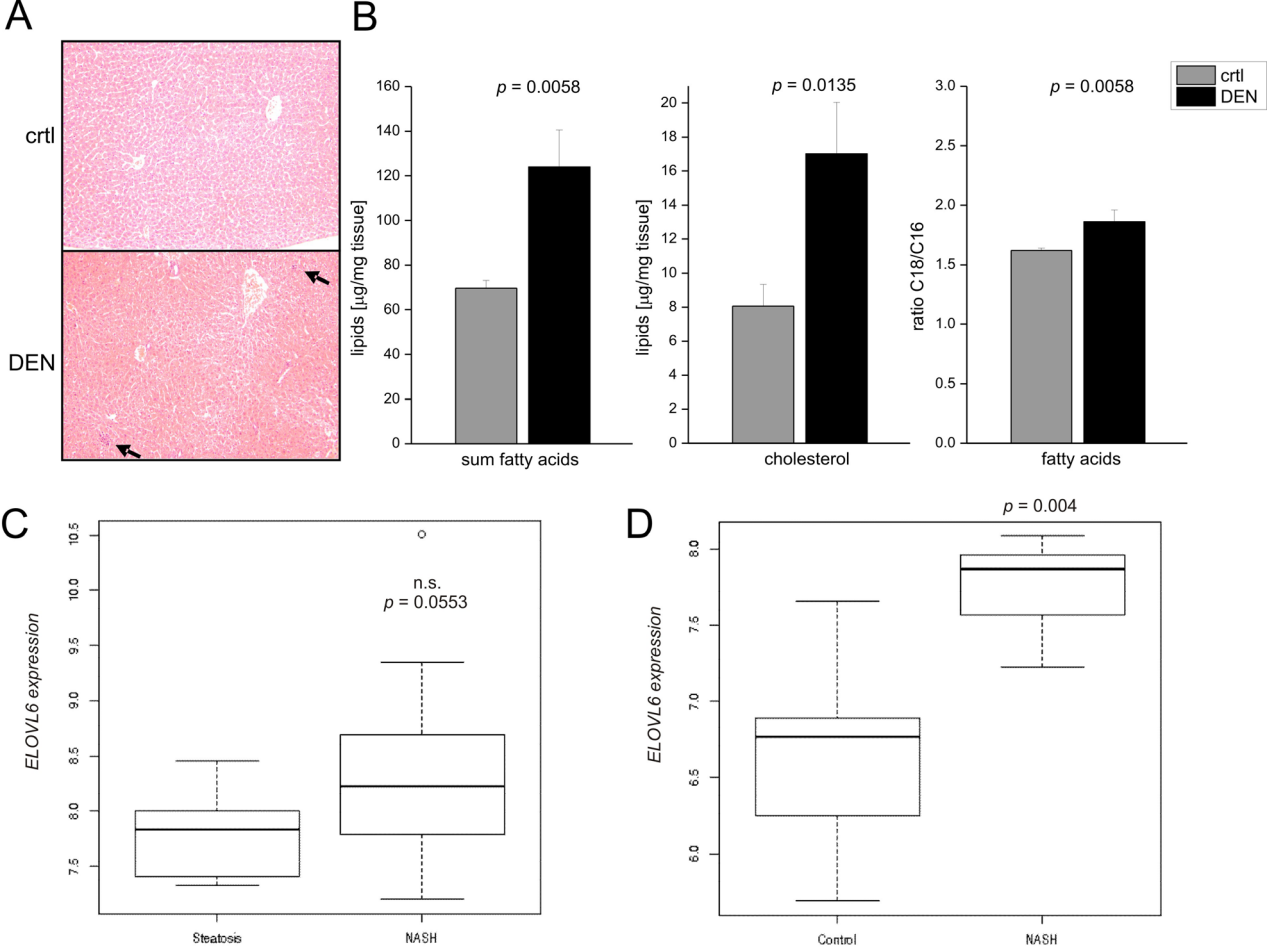

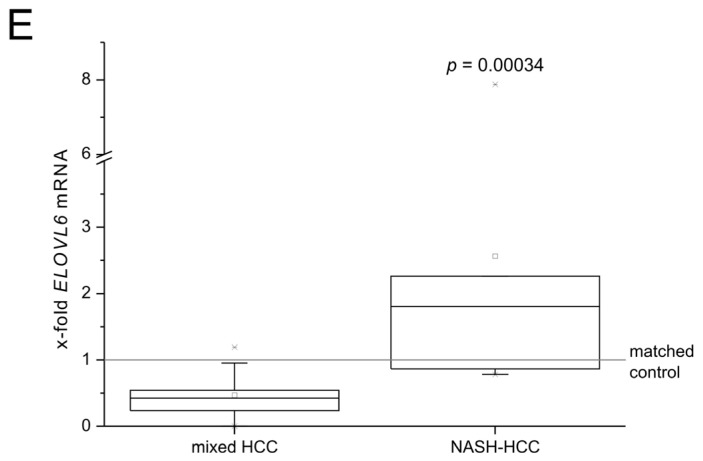
(**A**) Representative liver sections stained with HE from animals treated with DEN (DEN) compared to untreated control (crtl) (original magnification 200×). Arrows denote inflammatory foci; (**B**) Sum of all fatty acids, hepatic cholesterol, and ratio of C18/C16 fatty acids of DEN treated animals compared to untreated control (ctrl) are displayed (*n* = 6–15); (**C**,**D**) Expression of ELOVL6 in human NASH (*n* = 18) compared to steatosis (*n* = 14) (GSE48452) (n.s. = not statistically significant) (**C**) as well as healthy control samples (*n* = 8 for NASH; *n* = 7 for control; GSE37031) (**D**); and (**E**) *ELOVL6* mRNA expression in human NASH-related HCC samples (NASH-HCC) (*n* = 6) compared to HCC with mixed etiology (mixed HCC) (*n* = 26) [40]. Expression of tumor tissues was normalized to matched normal liver tissue (matched control).

To further investigate the role of fatty acid elongation in hepatocarcinogenesis, we used short-term (48 h) treatment with the carcinogen diethylnitrosamine (DEN) to model early inflammatory events associated with hepatocarcinogenesis [32,33]. In fact, we histologically observed inflammatory foci in the livers exposed to DEN (Figure 3A). Little is known about increased lipid accumulation after DEN treatment: Histologically detected hepatic lipid deposition by DEN was reported in fish (*Oryzias lapites*) [34,35]. Changes in the lipid composition have been reported for cancerous tissues compared to normal tissue in DEN-induced hepatocarcinogenesis [36–38], but not in the precancerous short-term protocol. We observed that inflammatory events were paralleled by distinct metabolic alterations similar to the murine NASH model: fatty acids, cholesterol levels, and the C18/C16 ratio were elevated upon 48 h DEN treatment (Figure 3B). This short-term model might therefore resemble NASH-related hepatocarcinogenesis. In later stages of DEN-induced carcinogenesis and during tumor progression we observed that fatty acid elongation was rather repressed [39]. This can be explained by the fact that the long-term DEN model predominantly acts via genotoxic effects of the carcinogen and therefore no NASH-related HCCs are induced.

In order to study the relevance of increased fatty acid elongation in human NASH and human NASH-related HCC, we analyzed the hepatic mRNA expression of the enzyme ELOVL6, which is responsible for the elongation of C16 fatty acids. We observed increased levels of *ELOVL6* in NASH *versus* steatosis samples (GSE48452 [41]) (Figure 3C) as well as in NASH compared to healthy control tissues (GSE37031 [42]) (Figure 3D). Interestingly, expression of *ELOVL6* was also altered in NASH-associated HCCs compared to HCC tissues of mixed etiology (Figure 3E): human NASH-related HCCs express increased levels of ELOVL6, indicating fatty acid elongation to play a critical role in this particular HCC subtype.

## 3. Experimental Section

### 3.1. Animals

All animal procedures were performed in accordance with the local animal welfare committee (#13/2009, 09/06/2009; #34/2010, 15/11/2010; Landesamt für Soziales, Gesundheit und Verbraucherschutz Saarland). Mice were kept under stable conditions regarding temperature, humidity, food delivery, and 12 h day/night rhythm. At the age of 3 weeks mice (DBA2/Bl6/J background) were fed either a methionine-choline deficient (MCD) or a methionine-choline supplemented control (crtl) diet for 3 weeks. Intraperitoneal clodronate or empty liposome injections [43] were started two days prior to MCD or control diet and repeated every five days to ensure Kupffer cell depletion. Leptin deficient mice *ob*/*ob* (Bl6:Cg-*Lep^ob^*/J) and lean control mice (*ob*/*+*) were obtained from Charles River and sacrificed at an age of 10 weeks. DEN treatment of mice (DBA2/Bl6/J background) on regular chow was performed by a single intraperitoneal injection of 100 mg/kg body weight at the age of 2.5 weeks. Mice were sacrificed 48 h after DEN injection. Animals of all experimental groups were sacrificed in a non-fasted state.

### 3.2. Human Liver Tissue

Paraffin-embedded liver samples from randomly selected pseudonymized HCC patients who underwent liver resection at the Saarland University Medical Center between 2005 and 2010 were obtained as described previously [40]. The study protocol was approved by the local Ethics Committee (#47/07). Samples had a mixed etiology including NASH, alcoholic liver disease, viral hepatitis, hemochromatosis, porphyria, and cryptogenic [40].

### 3.3. Fatty Acid Measurement by Gas Chromatography-Mass Spectrometry (GC-MS)

Murine liver samples were lyophilized and analyzed according to Bode *et al.* [44]. In short, lyophilized samples were dissolved in a mixture of 500 μL methanol/toluene/sulfuric acid (50:50:2, *v*/*v*/*v*) and incubated at 55 °C overnight. Subsequently, 400 μL of a 0.5 M NH_4_CO_3_, 2 M KCl solution were added and samples were centrifuged. The organic phase was transferred into a new glass vial, derivatized with 25 μL *N*-methyl-*N*-(trimethylsilyl)trifluoroacetamide at 37 °C for 1 h. Fatty acid separation was performed on an Agilent 6890N gas chromatograph coupled to an Agilent 5973N mass selective detector and equipped with a non-polar J&WDB-5HT capillary column (Agilent Technologies, Böblingen, Germany). The column temperature was kept at 130 °C for 2.5 min, increased to 240 °C at a rate of 5 °C/min, and then ramped to 300 °C at 30 °C/min, and held at 300 °C for 5 min. Helium was used as the carrier gas at a flow rate of 1 mL/min. The mass selective detector was operated in scan mode, average spectra were acquired in the *m*/*z* range of 40–700 *m*/*z* and were recorded at a scan speed of 2.24 scans/s. Scan control, data acquisition, and processing were performed by MSD ChemStation (Agilent Technologies, Böblingen, Germany) and AMDIS software based on the fragmentation patterns and retention time, in comparison with the reference standards Supelco 37 Component FAME Mix (Sigma-Aldrich, Taufkirchen, Germany), and NIST 08 library. Methyl-nonadecanoate (74208, Sigma-Aldrich, Taufkirchen, Germany) was used as an internal standard. The method detects both free and bound fatty acids.

### 3.4. Histochemistry and Immunohistochemistry

Hematoxylin-eosin staining of paraffin-embedded tissues was performed as previously reported [45,46]. Immunohistochemical F4/80 detection was achieved using the Vectastain Peroxidase Elite ABC kit/DAB with anti-F4/80 antibody (AbD Serotec, Puchheim, Germany) 1:1000 overnight at 4 °C. Epitopes were demasked with citrate buffer pH 6.0 for 10 min in a waterbath at 95 °C.

### 3.5. Analysis of the Public Gene Omnibus (GEO) Datasets

Datasets GSE48452 and GSE37031 [41,42] normalized using log2-RMA and log2-GCRMA respectively, were downloaded from Gene omnibus (GEO) [47]. Dataset GSE48452 with samples from different stages of NAFLD contained 18 NASH and 14 steatosis samples while dataset GSE37031 included 8 NASH and 7 control samples. The statistical significance was determined by Kolmogorov-Smirnov test.

### 3.6. Statistical Analysis

Results are expressed as means ± SEM. The statistical significance was determined by independent two-sample *t*-test. Expression data of human tissues were analyzed using Mann-Whitney *U* tests. The results were considered as statistically significant when *p* value was less than 0.05.

## 4. Conclusions

In the present study, we identified that NASH-induced fatty acid elongation is an inflammation-associated pathophysiological step in liver disease. Furthermore, the fatty acid elongase *ELOVL6* is elevated in human NASH and NASH-related HCC.
